# High-Throughput Sequence Analysis of Turbot (*Scophthalmus maximus*) Transcriptome Using 454-Pyrosequencing for the Discovery of Antiviral Immune Genes

**DOI:** 10.1371/journal.pone.0035369

**Published:** 2012-05-18

**Authors:** Patricia Pereiro, Pablo Balseiro, Alejandro Romero, Sonia Dios, Gabriel Forn-Cuni, Berta Fuste, Josep V. Planas, Sergi Beltran, Beatriz Novoa, Antonio Figueras

**Affiliations:** 1 Instituto de Investigaciones Marinas, IIM, CSIC, Vigo, Spain; 2 Departament de Fisiologia i Immunologia, Facultat de Biologia, Universitat de Barcelona i Institut de Biomedicina de la Universitat de Barcelona, IBUB, Barcelona, Spain; 3 Centros Científicos y Tecnológicos de la UB, CCiT-UB, Universitat de Barcelona, Edifici Clúster, Parc Científic de Barcelona, Barcelona, Spain; Auburn University, United States of America

## Abstract

**Background:**

Turbot (*Scophthalmus maximus* L.) is an important aquacultural resource both in Europe and Asia. However, there is little information on gene sequences available in public databases. Currently, one of the main problems affecting the culture of this flatfish is mortality due to several pathogens, especially viral diseases which are not treatable. In order to identify new genes involved in immune defense, we conducted 454-pyrosequencing of the turbot transcriptome after different immune stimulations.

**Methodology/Principal Findings:**

Turbot were injected with viral stimuli to increase the expression level of immune-related genes. High-throughput deep sequencing using 454-pyrosequencing technology yielded 915,256 high-quality reads. These sequences were assembled into 55,404 contigs that were subjected to annotation steps. Intriguingly, 55.16% of the deduced protein was not significantly similar to any sequences in the databases used for the annotation and only 0.85% of the BLASTx top-hits matched *S. maximus* protein sequences. This relatively low level of annotation is possibly due to the limited information for this specie and other flatfish in the database. These results suggest the identification of a large number of new genes in turbot and in fish in general. A more detailed analysis showed the presence of putative members of several innate and specific immune pathways.

**Conclusions/Significance:**

To our knowledge**,** this study is the first transcriptome analysis using 454-pyrosequencing for turbot. Previously, there were only 12,471 EST and less of 1,500 nucleotide sequences for *S. maximus* in NCBI database. Our results provide a rich source of data (55,404 contigs and 181,845 singletons) for discovering and identifying new genes, which will serve as a basis for microarray construction, gene expression characterization and for identification of genetic markers to be used in several applications. Immune stimulation in turbot was very effective, obtaining an enormous variety of sequences belonging to genes involved in the defense mechanisms.

## Introduction

Turbot (*Scophthalmus maximus*) is an economically important flatfish species belonging to the family *Scophthalmidae* (order *Pleuronectiformes*) widely distributed from Norway to the Mediterranean and the Black Sea [1]. Nowadays, the culture of this fish is well-established and a full-cycle aquaculture is in place. In Europe, turbot aquaculture production was 9,067 tons in 2008, representing almost 90% of total flatfish production, being Spain the main European producer [2]. This species, native to Europe, is also cultured in China, reaching an annual level of 50,000–60,000 tons in recent years and being the largest producer of turbot in the world [3].

This development caused a parallel increase in pathological conditions affecting this culture. Several pathogens, including bacteria [4], virus [5] and parasites [6] affect the health status of the farmed fish causing important economic losses. In spite of the relevance of turbot culture and the associated pathological process, knowledge about its immune system is still fragmentary and little is known about host-pathogen interactions. The pathways implicated in the response against pathogens remain incomplete in fish and the understanding of how those defense mechanisms act is a relevant factor in order to enhance resistance of cultured fish to diseases. The identification of immune-relevant genes, that could be potential markers for disease resistance, is a step to establish a successful genetic breeding program. Moreover, several immune molecules (antimicrobial peptides and cytokines) could be used as novel anti-infective drugs and also as adjuvants in vaccination processes due to their immunomodulatory properties [7]–[14]. In addition, increase in immune-related genes and pathways in turbot could help us to improve the microarray tools available for research in fish immunology.

Expressed sequence tags (ESTs) are short sequence reads derived from cDNA libraries that represent a useful tool for discovery and identification of transcripts mainly in species without a fully sequenced genome [15], [16]. All published approaches to the turbot immune transcriptome are based on classical cloning and Sanger sequencing strategies [17]–[19]. Pyrosequencing is a relatively new sequencing technology in fish biology, which provides a fast and cost-effective way to generate large amounts of data from non-model species such as turbot.

The aim of this work was to increase the genomic resources of turbot, particularly the transcriptome in response to viral stimulations and to identify the main components of the immune pathways. For this, we have used 454-pyrosequencing on several turbot tissues stimulated with two different viruses (viral haemorrhagic septicaemia virus and nodavirus), plasmid constructs used as DNA vaccines and polyriboinosinic polyribocytidylic acid (poly I:C). The data generated in this high-throughput sequence analysis will serve as a basis for microarray construction, which will also be useful for further research on this important aquaculture species.

## Results and Discussion

### Transcriptomic Analysis

**Figure 1:**
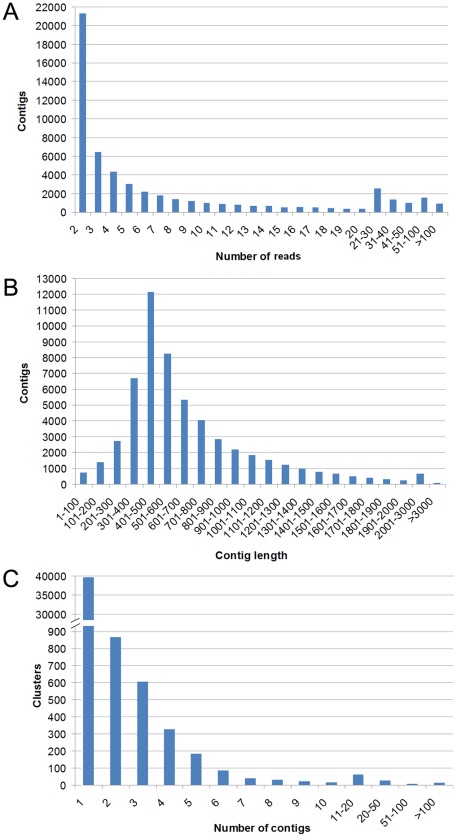
*Scophthalmus maximus* transcriptome assembly statistics. (A) Distribution of number of reads per contig in the normalized library. (B) Size distribution of 454 sequences after contig construction. (C) Distribution of cluster composition by contigs.

Table 1 shows the summarized statistics for Roche 454-pyrosequencing of turbot normalized library. Sequencing of turbot transcriptome yielded a total of 915,782 reads totaling 291.04 megabases with an average read length of 318 bp. After processing the reads and filtering the low-quality and small ones, 915,256 high quality reads with an average size of 297 bp were submitted to MIRA V3.2.0 for assembly. Reads obtained in this high-throughput sequence analysis were submitted to the NCBI Short Read Archive (http://trace.ncbi.nlm.nih.gov/Traces/sra/sra.cgi) under accession number SRA046015.2. 733,411 reads were assembled into 55,404 contigs, with an average contig length of 671 bp. Moreover, contigs generated in this work are available on the NCBI Transcriptome Shotgun Assembly Sequence Database (http://www.ncbi.nlm.nih.gov/genbank/tsa): BioProjectID PRJNA89091 (Genbank accessions JU351362 to JU404686). In order to minimize redundancy, contigs were grouped into a total of 41,870 clusters according to their sequence similarity. Of 55,404 contigs, 24,845 (44.84%) had a significant BLASTx hit to proteins present in Swissprot/Metazoan Refseq/nr databases and matched 12,591 unique protein accessions. The descriptive analysis presented here utilized only the contigs produced by the assembly. Contig and clustering construction are summarized in Figure 1. Singletons (181,845 sequences) were separately analyzed and annotated for the search of new sequences showing homology to proteins involved in the main immune signaling cascades but were not included in the other analysis in order to obtain more reliable results. Only 13,722 singletons were successfully annotated. An additional file containing the singletons selected for their annotation is also provided (Table S1). Before starting our analysis there were only 12,471 EST and less of 1,500 nucleotide sequences for *S. maximus* in NCBI database. Other approaches to increase the knowledge on the turbot immune transcriptome had been previously conducted using strategies based in Sanger sequencing. Wang et al. [17] obtained 49 ESTs from kidney and spleen of turbot following challenge with *Vibrio harveyi*. Pardo et al. [18] achieved 1,073 contigs and 2,409 singletons from 9,256 ESTs from liver, spleen and head kidney of turbot infected with *Aeromonas salmonicida, Philasterides dicentrarchi* and from healthy fish. Park et al. [19] obtained 3,173 ESTs from liver, kidney and gill tissues of nodavirus-infected turbot. Pyrosequencing represents a step forward compared to classical Sanger sequencing strategies and allows to generate great amounts of genomic and transcriptomic information at relatively low cost and in short periods of time. The present work increases dramatically the number of putative transcripts by providing 55,404 contigs for further genomic studies in turbot and represents the most effective attempt to improve the knowledge of *S. maximus* transcriptome. Moreover, it was possible to annotated 24,845 of these contigs (44.84%) with an E value cut off of 1e-3 after Blastx to selected databases. This relatively low value of annotation is almost certainly due to the scarce information available in the database for pleuronectiform fish.

**Table 1:** Summary statistics of *Scophthalmus maximus* 454-pyrosequencing.

Data generation and filtering	
Number of total reads	915,782
Total Megabases	291.04
Average read length (bp)	317.8
N50 read length (bp)	396
Number of high quality reads (after filtering)	915,256 (99.94%)
Total Megabases (after filtering)	271.95
Average read length (after filtering)	297.14
N50 read length (after filtering)	381
**Assembly statistics**	
Number of reads assembled	733,411 (80.13%)
Number of contigs	55,404
Total consensus Megabases	37.2
Average contig coverage	4.4
Average contig length	671.3
N50 contig length	756
Range contig length	40–6,821
Number of contigs >500 pb	31,764 (57.33%)
Number of contigs with 2 reads	21,270 (38.39%)
Number of contigs with >2 reads	34,134 (61.61%)
Number of clusters	41,870
Number of clusters with 1 contig	39,578 (94.53%)
Number of clusters with >1 contig	2,292 (5.47%)
Number of singletons	181,845
**Annotation**	
Total number of annotated contigs	24,845 (44.84%)
SwissProt+Metazoan RefSeq hits	20,175 (81.2%)
nr hits	4,670 (18.8%)
Unique protein hits	12,591
Total number of annotated singletons	13,722

**Figure 2:**
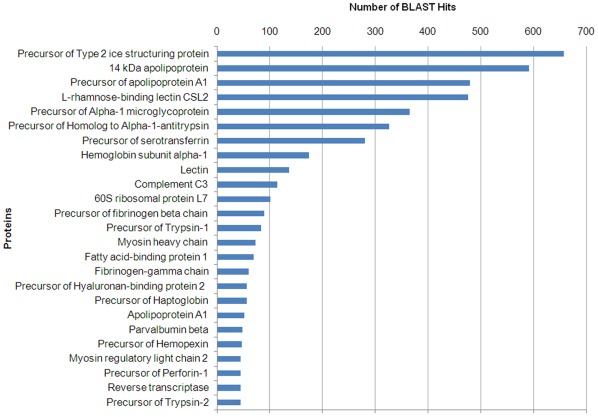
BLASTx top-hit sequence distribution of gene annotations. Y-axis represents the most abundant annotation terms for the contigs obtained in the 454-pyrosequencing. X-axis represents the number of contigs presenting each annotation term.

A top-25 showing the most commonly detected proteins terms in the annotation process represented different functional groups including an elevated amount of immune-related proteins (Figure 2). The precursor of type 2 ice structuring protein was surprisingly the more represented BLAST hit (654 hits). Antifreeze proteins (AFPs) have in common the ability to bind to ice and inhibit its growth [20]. Type II antifreeze proteins found in smelt (*Osmerus mordax*) and herring (*Clupea harengus harengus*) belong to the C-type lectin superfamily, although these ice-binding proteins do not appear to function as lectins [21]. Millán et al. [22] using an oligo-microarray specific for turbot, found a great up-regulation of antifreeze polypeptide precursor in fish challenged intraperitoneally with the bacteria *Aeromonas salmonicida*. It could be interesting to study whether this molecule possesses any antiviral function. Lectins are recognition molecules capable to adhere to carbohydrates that are implicated in direct first-line defense against pathogens [23]. L-rhamnose-binding lectin CSL2 (476 hits) and lectin (137 hits) also appear represented in the BLASTx top-hit sequences.

Other immune-related proteins appearing in the top-hit sequences are 14 kDa apolipoprotein (591 hits), precursor of apolipoprotein A1 (480 hits) and apolipoprotein A1 (52 hits). Apolipoproteins with a molecular weight of 14 kDa (apo-14 kDa) are associated with fish plasma high density lipoproteins (HDL) and phylogenetic analysis indicates that fish apo-14 kDa can be the homologue of mammalian apoA-II [24]. Although the main role of HDL/apolipoproteins seems to be its participation in cholesterol transport [25], multiple immune functions have been reported for mammalians and fish, including antiviral, antibacterial and anti-inflammatory activities [26]–[32].

Both precursor of alpha-1 microglycoprotein (365 hits) and precursor of homolog to alpha-1-antitrypsin (326 hits) possess an immunomodulatory role regulating the inflammation process [33]–[35]. Precursor of trypsin-1 (83 hits) and precursor of trypsin-2 (45 hits) are serine proteases presenting a proinflammatory role during the pathogenesis [36], [37] besides their digestive function.

Other group widely represented was related with the complement cascade (complement component C3 with 114 hits) and coagulation (precursor of fibrinogen beta chain with 90 hits and fibrinogen-gamma chain with 60 hits). Fibrinogen is important not only for its pro-coagulatory functions but also for its pro-inflammatory activities [38], [39]. Precursor of Hyaluronan-binding protein 2 (56 hits) and other hyaluronan related proteins regulate inflammation and tissue damage through the cell recruitment and release of inflammatory cytokines [40].

Precursor of serotransferrin (280 hits) is a glycoprotein with iron-chelating properties that transports and scavenges extracellular iron [41]. Precursor of haptoglobin (56 hits) and precursor of hemopexin (47 hits) are acute phase proteins that recycle hemoglobin from senescent erythrocytes [42], [43]. Moreover, Hemoglobin subunit alpha-1 (174 hits) was another sequence belonging to the top-hit sequences. The high transcriptional level of these genes could be due to the anemia and hemorrhaging caused by viral haemorrhagic septicaemia virus (VHSV) [44] and also to the ability of the viruses to maintain an iron-replete host for replication [45].

Furthermore, Precursor of Perforin-1 (45 hits) is a molecule implicated in the cellular lysis through the formation of pores in biological membranes [46]. The other sequences represented in the top-hit BLASTx are mainly implicated in transport, motor activity and transcription. The large presence of immune-related terms in this ranking revealed a very effective stimulation of turbot and a successful transcriptome sequencing.

### Gene Ontology Analysis

**Figure 3:**
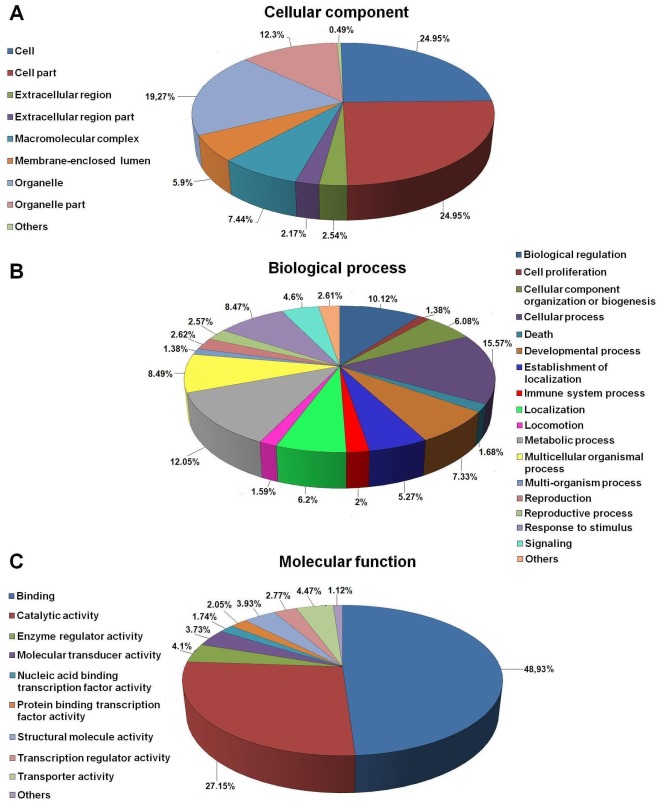
Gene ontology (GO) assignment (2^nd^ level GO terms) for the transcriptomic sequences (contigs) of *Scophthalmus maximus*. (A) Cellular component, (B) Biological process and (C) Molecular function. Numbers refer to percentage of occurrences.

Gene ontology (GO) is commonly used to categorize gene products and standardize their representation across species [47]. In order to eliminate redundancy and given that the contigs belonging to the same cluster present the same annotation, only the largest contig of each cluster was submitted to *Blast2GO* suite [48] for the assignment to three functional groups based on GO terminology: Cellular Component, Biological Process and Molecular Function. 12,534 contigs (29.9%) were assigned to a GO category. Figure 3 summarizes GO terms at 2^nd^ level. Cellular component terms (Figure 3A) showed a significant percentage of clusters assigned to cell (24.95%) and cell part (24.95%), whereas 19.27% were related to organelle and 12.3% to organelle part. The most represented biological process terms (Figure 3B) were related to cellular process (15.57%), metabolic process (12.05%) and biological regulation (10.12%), suggesting a high degree of metabolic activity of the sampled tissues. Immune-related proteins could be included within cellular process category (which includes the molecules implicated in cell activation), death (1.68%), immune system process (2%), multicellular organismal process (8.49%) (which includes proteins related to the coagulation process), response to stimulus (8.47%) and signaling (4.9%). Finally, within the molecular function category (Figure 3C), 48.93% were related to binding (including several immune-processes) followed by 27.15% related to catalytic activity. Our GO analysis followed a similar pattern to that obtained in previous pyrosequencing efforts performed in other fish species [49]–[51]. In order to provide a more detailed analysis of the Molecular Function category an additional file showing GO terms at the 3^rd^ level is also supplied (Table S2).

### Taxonomic Analysis

**Figure 4:**
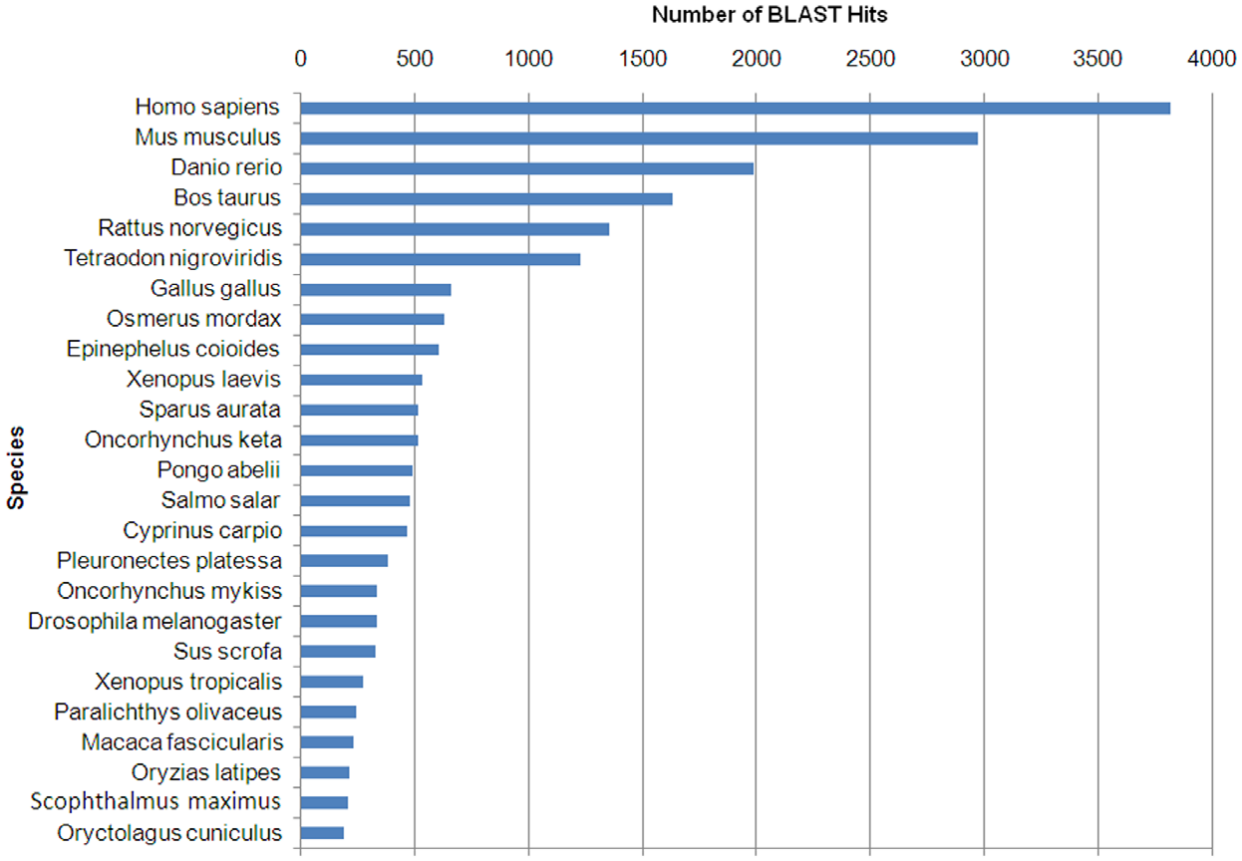
BLASTx top-hit species distribution of gene annotations. Y-axis represents the species presenting more match hits with the contigs obtained in the 454-pyrosequencing. X-axis represents the number of contigs presenting protein match hits to these species.

A BLASTx top-hit species distribution of gene annotations showed highest homology to *Homo sapiens*, followed by *Mus musculus* (Figure 4). These results could be explained by the elevated presence of human and murine sequences in the databases. The third position was for *Danio rerio*, which has been widely used as a model organism for biomedical and fish studies. Other species with known genome sequences appearing in the BLASTx top-hit were the mammals *Bos taurus* and *Rattus norvergicus*, the chicken *Gallus gallus* and the fruit fly *Drosophila melanogaster*. There were 13 fish species among the top-hits, but only 3 species belonging to the order *Pleuronectiformes*. Turbot appeared in position 24 and only 0.85% of the BLASTx top-hits matched *Scophthalmus maximus* protein sequences. This may be explained on the basis of the limited number of turbot proteins (605) currently available in the NCBI database compared to other fish species.

**Figure 5:**
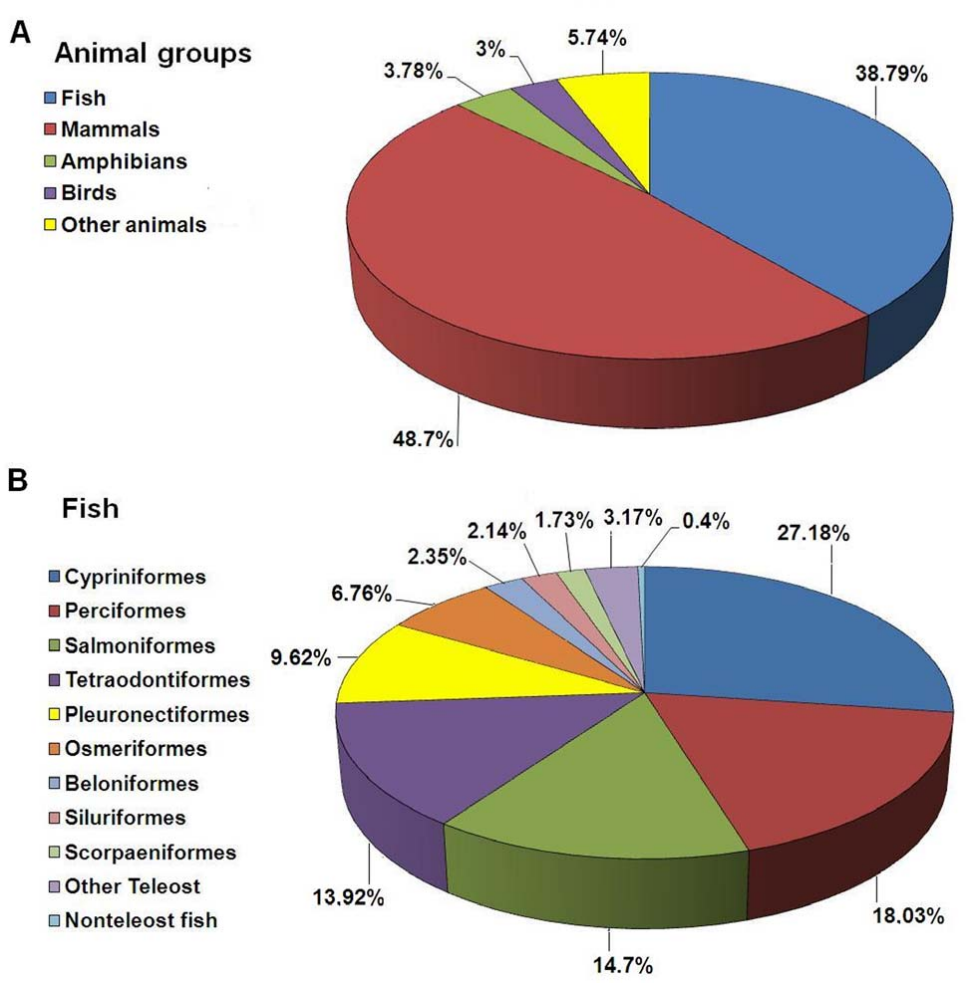
Pie chart showing species distribution of the BLAST hits of the *Scophthalmus maximus* sequences. (A) Distribution of the BLAST hits within the kingdom *Animalia*. (B) Distribution of the BLAST hits among teleost orders.

A more detailed taxonomic analysis was performed in order to further characterize our pyrosequencing data. The immense majority of the annotated sequences matched organisms from the kingdom *Animalia* (97.8%), but there was representation from all other kingdoms, as well as from viruses. In *Animalia*, mammals represented 48.7% of the hits, whereas fish represented 38.79% (Figure 5A). This suggests that we have identified a large number of genes poorly described in fish. The teleost orders most represented in the annotation results were *Cypriniformes* (27.18%), *Perciformes* (18.03%), *Salmoniformes* (14.7%) and *Tetraodontiformes* (13.92%) (Figure 5B). These orders include model fish species and organisms with economic relevance and, consequently, with abundant transcriptomic information available. The order *Pleuronectiformes* represented only 9.62% of the total hits from fish, highlighting once again the scarce information available for this group in the databases and reaffirming the need to improve and increase the information. The present massive transcriptome analysis has helped to solve this problem. The kingdom *Plantae* represented 0.6% of annotated sequences, *Fungi* 0.446%, *Protista* 0.43%, *Eubacteria* 0.462%, *Archaebacteria* 0.045% and Viruses 0.118%. Despite water treatment at our facilities with filters and UV light it is possible that certain microorganisms such as algae, fungi, protozoa and bacteria have survived these conditions and have contaminated the samples. Moreover, the normal microbiota of turbot tissues could be present. However, these contigs showing homology to non-animal species represent a negligible percentage of the total annotated contigs. Among bacteria, some sequences seemed to show homology to proteins from genera that include relevant pathogens for fish species: *Vibrio*, *Pseudomonas* and *Mycobacterium*. Among viruses, there was also homology to proteins from viral families that can affect fish: *Herpesviridae*, *Retroviridae* and *Rhabdoviridae*. The *Rhabdoviridae* VHSV, which was used to inoculate turbot in our study, appeared represented in the annotation.

### Comparative Analysis

**Figure 6:**
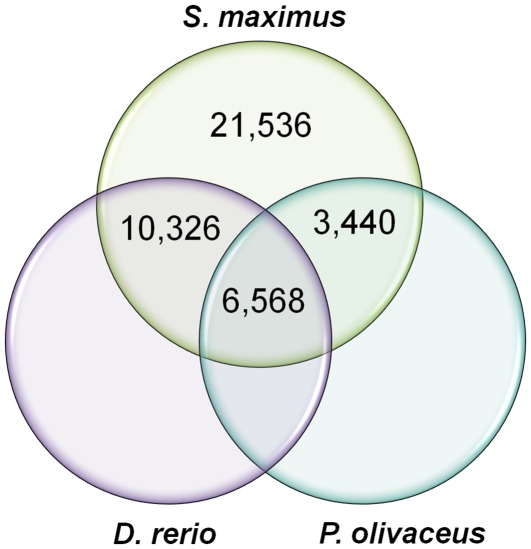
Venn diagram showing the comparison among *Scophthalmus maximus* transcriptomic sequences with the known sequences from *Danio rerio* and *Paralichthys olivaceus* deposited in the NCBI database.

The turbot transcriptomic sequences were compared to ESTs and nucleotide sequences from zebrafish (*D. rerio,* order *Cypriniformes*) and Japanese flounder (*Paralichthys olivaceous,* order *Pleuronectiformes*) present in the NCBI database in order to detect the presence of novel proteins in turbot remaining unknown in other fish species more studied so far. The results are displayed as a Venn diagram in Figure 6. Zebrafish was chosen because it represents a model fish and its genome has been sequenced. Japanese flounder was chosen because it is a species phylogenetically close to turbot and with abundant genetic information compared to other flatfish. The comparison of our pyrosequencing results with the information available in databases for two species presenting a better genomic and transcriptomic knowledge can give an idea of the new contributions to the fish transcriptome study. Of the total 41,870 contigs representing each cluster, 21,536 (51.44%) had no significant similarity to any protein identified within the sequences of *D. rerio* and *P. olivaceus*. This high percentage of unique transcripts can be attributed to the presence of novel genes but also it can be derived from untranslated regions (5′ and 3′ UTR) and non-conserved areas of proteins [52], [53]. 10,326 contigs (24.66%) were shared between zebrafish and turbot but not with Japanese flounder, possibly due to the high number of sequences present in the NCBI database for this model species. 3,440 sequences (8.22%) were shared between Japanese flounder and turbot but not with zebrafish, suggesting that they are potential flatfish-specific sequences. However, 6,568 contigs (15.69%) had homology with both *D. rerio* and *P. olivaceus* sequences and probably represent well conserved genes across the species.

### Immune Transcriptome Analysis

Our high-throughput sequencing effort revealed the presence of an elevated number of relevant molecules for the immune response, but hitherto remaining unknown in *S. maximus*. There were no evidences of existence of the majority of the detected immune-related proteins in turbot before performing this pyrosequencing analysis. Here, we describe the main components of the immune pathways identified in the *Scophthalmus maximus* transcriptome (contigs and singletons).

#### Complement pathway

**Figure 7:**
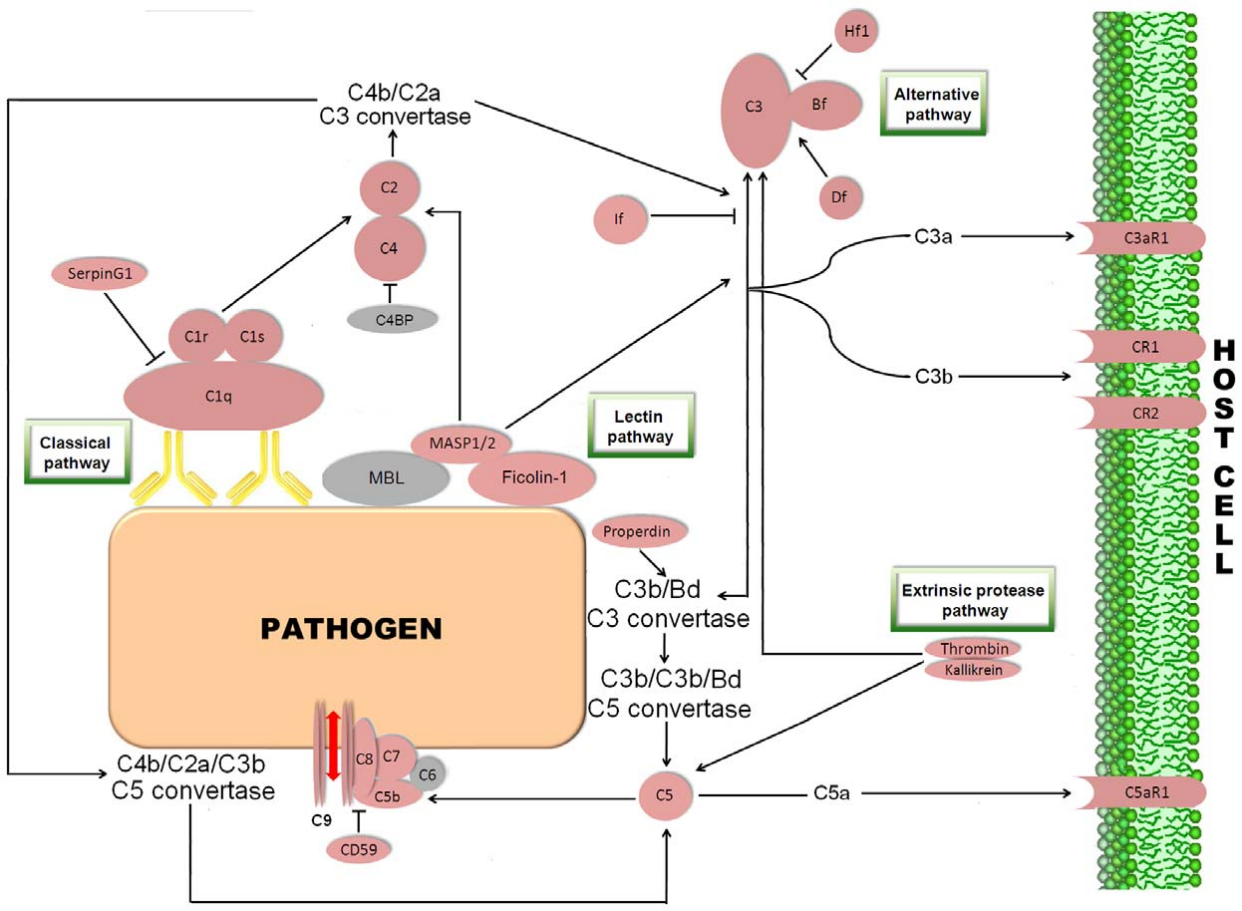
Complement pathway representing the present and absent proteins in the pyrosequencing results. Proteins appearing in the turbot transcriptome are represented in reddish colour and absent proteins in grey color. C1q: complement C1q subcomponent; C1r: complement C1r subcomponent; C1s: complement C1s subcomponent; C2: complement component 2; C3: complement component 3; C4: complement component 4; C5: complement component 5; C6: complement component 6; C7: complement component 7; C8: complement component 8; C9: complement component 9; C4BP: complement component 4 binding protein; MBL: mannose-binding lectin; MASP1/2: mannan-binding lectin serine protease 1/2; Bf: component factor B; Hf1: complement factor H; Df: component factor D; If: complement factor I; C3aR1: C3a anaphylatoxin chemotactic receptor; CR1: complement component receptor 1; CR2: complement component receptor 2; C5aR1: C5a anaphylatoxin chemotactic receptor.

The complement system is a biochemical cascade involving more than 35 soluble plasma proteins. This defense mechanism is essential in the innate immunity, but it exhibits also the ability to stimulate the specific immune response [54]. In vertebrates, the central component C3 is proteolytically activated by a C3 convertase through the classic, lectins and alternative routes [55]. In the present study, we identified the main putative central molecules in the complement system (C1 to C9) (Figure 7). Only the C6 component appeared not to be represented. C2, C4 and C5 were identified for the first time in turbot, as well as the receptors for C3a (C3aR1), C5a (C5aR1), the complement receptor type 1 or C3b/C4b receptor (CR1) and the complement receptor type 2 (CR2 or CD21). Moreover, other important proteins intervening in the complement cascade were annotated, including the complement factors H (Hf), B (Bf), D (Df), I (If) and P (Pf) or properdin. Only one of these, Bf, had been reported previously in *S. maximus* [19]. The elevated number of complement components detected in turbot exposed to viral stimulation could reflect the pivotal importance of these molecules in protection during viral infections. An additional table containing information about contigs and singletons showing homology to molecules implicated in the complement pathway is provided (Table S3).

#### Toll-like receptor signaling pathway

**Figure 8:**
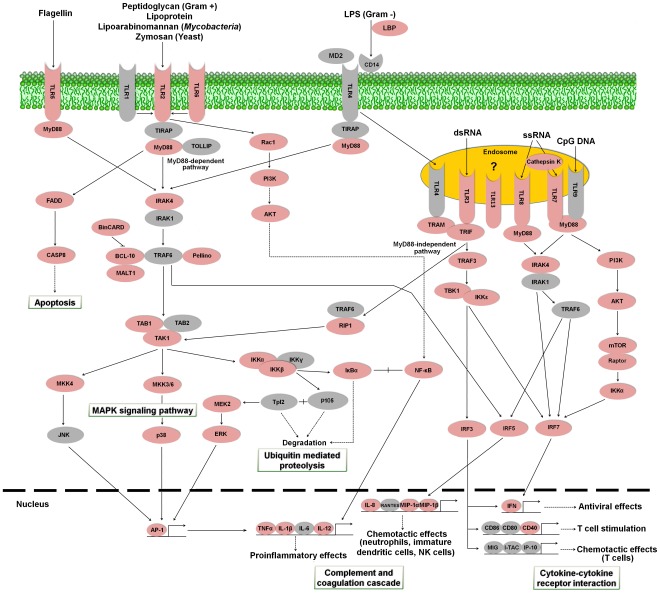
Toll-like receptor signaling pathway representing the present and absent proteins in the pyrosequencing results. Proteins appearing in the turbot transcriptome are represented in reddish color and absent proteins in grey color. LBP: lipopolysaccharide-binding protein; MD2: myeloid differentiation factor 2; TLR1: toll-like receptor 1; TLR2: toll-like receptor 2; TLR3: toll-like receptor 3; TLR4: toll-like receptor 4; TLR5: toll-like receptor 5; TLR6: toll-like receptor 6; TLR7: toll-like receptor 7; TLR8: toll-like receptor 8; TLR9: toll-like receptor 9; TLR13: toll-like receptor 13; CD14: cluster of differentiation antigen 14; MyD88: myeloid differentiation primary response protein MyD88; TIRAP: toll-interleukin 1 receptor domain-containing adaptor protein; TOLLIP: toll-interacting protein; Rac1: Ras-related C3 botulinum toxin substrate 1; PI3K: phosphatidylinositol-4,5-bisphosphate 3-kinase; AKT: Rac serine/threonine-protein kinase; FADD: FAS-associated via death domain; IRAK1: interleukin-1 receptor-associated kinase 1; IRAK4: interleukin-1 receptor-associated kinase 4; TRAM: TRIF-related adaptor molecule; BinCARD: BCL-10-interacting protein with CARD; BCL-10: B cell lymphoma 10; MALT1: mucosa associated lymphoid tissue lymphoma translocation gene; CASP8: caspase 8; TRAF: TNF receptor-associated factor 3; TRAF6: TNF receptor-associated factor 6; TAB1: TAK1-binding protein 1; TAB2: TAK1-binding protein 2; TAK1: TGF-beta activated kinase 1; RIP1: receptor-interacting serine/threonine-protein kinase 1; MKK3/6: mitogen-activated protein kinase kinase 3/6; MKK4: mitogen-activated protein kinase kinase 4; JNK: c-Jun N-terminal kinase; p38: p38 MAP kinase; NF-κB: nuclear factor NF-kappa-B; IKKα: inhibitor of nuclear factor kappa-B kinase subunit alpha; IKKβ: inhibitor of nuclear factor kappa-B kinase subunit beta; IKKγ: inhibitor of nuclear factor kappa-B kinase subunit gamma; IKKε: inhibitor of nuclear factor kappa-B kinase subunit epsilon; IκBα: NF-kappa-B inhibitor alpha; p105: nuclear factor NF-kappa-B p105 subunit; Tpl2: tumor progression locus 2; MEK2: mitogen-activated protein kinase kinase 2; ERK: extracellular signal-regulated kinase; TRIF: toll-like receptor adapter molecule 1; TBK1: TANK-binding kinase 1; mTOR: mechanistic target of rapamycin; Raptor: regulatory associated protein of mTOR; IRF3: interferon regulatory factor 3; IRF5: interferon regulatory factor 5; IRF7: interferon regulatory factor 7; AP-1: activator protein-1; TNFα: tumor necrosis factor alpha; IL-1β: interleukin 1 beta; IL-6: interleukin 6; IL-12: interleukin 12; IL-8: interleukin 8; RANTES: regulated on Activation. Normal T-cell Expressed and Secreted; MIP-1α: macrophage inflammatory protein-1α; MIP-1β: macrophage inflammatory protein-1β; IFN: interferon; CD86: cluster of differentiation antigen 86; CD80: cluster of differentiation antigen 80; CD40: cluster of differentiation antigen 40; MIG: monokine induced by gamma interferon; I-TAC: interferon-inducible T-cell alpha chemoattractant; IP-10: interferon-gamma-inducible protein 10.

Recognition of pathogen-associated molecular patterns (PAMPs) is essential for the activation of innate immunity and it is carried out by Toll-like receptors (TLRs). Cell activation triggered by TLR entails the activation of nuclear factor–κB (NF-κB) inducing pro-inflammatory cytokines through MyD88 dependent or independent pathways [56], [57]. We have found numerous contigs presenting high similarity to several members of the TLR cascade (Figure 8). So far in turbot, there was only evidence for the presence of two Toll-like receptors: TLR3 (GenBank accession FJ009111) and TLR11 [18]. We have identified several sequences with similarity to four new TLRs in *S. maximus*: TLR2, TLR5, TLR6, TLR7, TLR8, TLR13 and TLR21B. Moreover, most of the central proteins belonging to the turbot TLR signaling pathway have been discovered for the first time in this transcriptome analysis: MyD88, interleukin-1 receptor-associated kinase 4 (IRAK4), IκB kinase (IKK), NF-κB, and several mitogen-activated protein kinases (MAPKs) amongst others. An additional table containing information about contigs and singletons showing homology to molecules implicated in the Toll-like receptor signaling pathway is provided (Table S4).

#### B cell receptor signaling pathway

**Figure 9:**
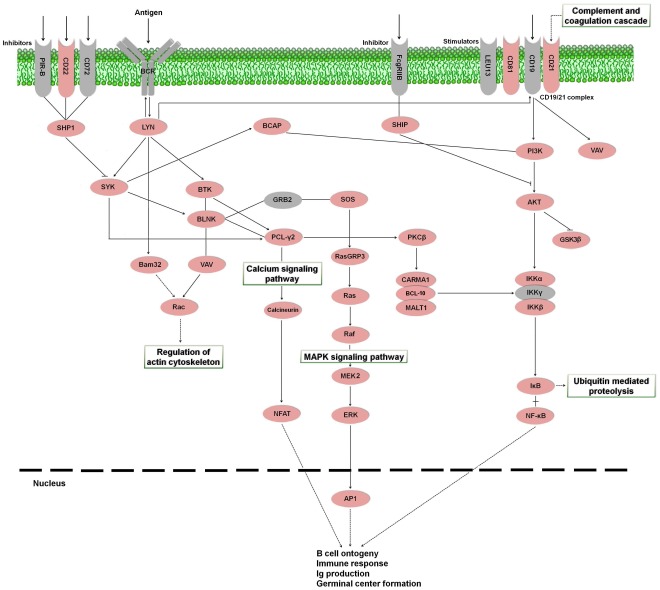
B cell receptor signaling pathway representing the present and absent proteins in the pyrosequencing results. Proteins appearing in the turbot transcriptome are represented in reddish color and absent proteins in grey color. PIR-B: paired-immunoglobulin like receptor-B; CD22: cluster of differentiation antigen 22; CD72: cluster of differentiation antigen 72; BCR: B cell receptor; FcgRIIB: Fc fragment of IgG, low affinity IIb, receptor; LEU13: leukocyte surface antigen LEU-13; CD81: cluster of differentiation antigen 81; CD19: cluster of differentiation antigen 19; CD21: cluster of differentiation antigen 21; SHP1: protein-tyrosine phosphatase-1; LYN: tyrosine-protein kinase Lyn; BCAP: B-cell phosphoinositide 3-kinase adapter protein; SHIP: SH-2-containing Inositol 5' Phosphatase; PI3K: phosphatidylinositol-4,5-bisphosphate 3-kinase; VAV: vav guanine nucleotide exchange factor; SYK: spleen tyrosine kinase; BTK: Bruton agammaglobulinemia tyrosine kinase; BLNK: B-cell linker; GRB2: growth factor receptor-binding protein 2; SOS: son of sevenless; PCL-γ2: phospholipase C gamma 2; PKCβ: protein kinase C beta; AKT: Rac serine/threonine-protein kinase, GSK3β: glycogen synthase kinase 3 beta; Bam32: B-cell adapter molecule of 32 kDa; Rac: Ras-related C3 botulinum toxin substrate 1; Ras: GTPase HRas; RasGRP3: Ras guanyl-releasing protein 3; CARMA1: caspase recruitment domain family, member 11; BCL-10: B cell lymphoma 10; MALT1: mucosa associated lymphoid tissue lymphoma translocation gene; IKKα: inhibitor of nuclear factor kappa-B kinase subunit alpha; IKKβ: inhibitor of nuclear factor kappa-B kinase subunit beta; IKKγ: inhibitor of nuclear factor kappa-B kinase subunit gamma; Raf: Raf proto-oncogene serine/threonine-protein kinase; MEK2: mitogen-activated protein kinase kinase 2; NFAT: nuclear factor of activated T-cells; ERK: extracellular signal-regulated kinase; IκB: NF-kappa-B inhibitor beta; NF-κB: nuclear factor NF-kappa-B; AP1: activator protein-1.

B lymphocytes and T lymphocytes are the main cells involved in antigen-specific defense. B cells are responsible for the production of specific antibodies that can neutralize pathogens [58]. The activation of B lymphocytes is achieved through the binding of the antigen to B-cell receptors [59]. Prior to the present study, there was only genetic and bibliographic information available for turbot immunoglobulins (Ig) (GenBank accessions FJ617006, DQ848956, DQ848958, AJ296096; [19]), Ras-related C3 botulinum toxin substrate 1 (Rac1) (FJ361904), Ras GTPase (EU711052), extracellular signal-regulated kinase (ERK) [19] and interferon induced transmembrane protein (Leu13 or CD225) (DQ848974). We have detected in *S. maximus* transcriptome the majority of the proteins implicated in the B cell signaling pathway in mammals (Figure 9). An additional table containing information about contigs and singletons showing homology to molecules involved in the B cell receptor signaling pathway is also provided (Table S5).

#### T cell receptor signaling pathway

**Figure 10:**
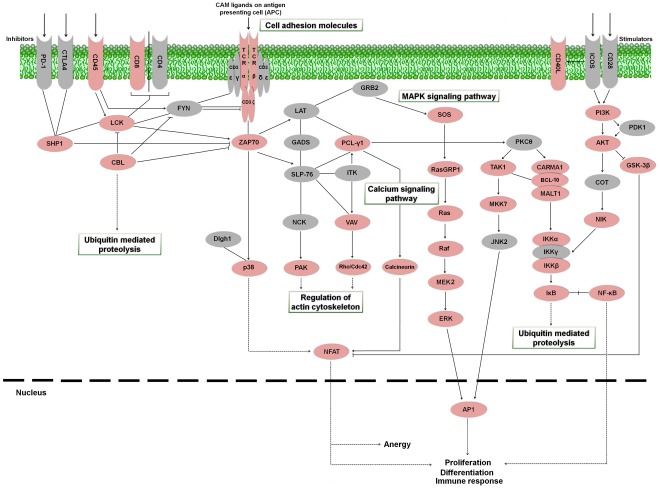
T cell receptor signaling pathway representing the present and absent proteins in the pyrosequencing results. Proteins appearing in turbot the transcriptome are represented in reddish color and absent proteins in grey color. PD-1: programmed cell death protein 1; CTLA4: cytotoxic T-lymphocyte-associated protein 4; CD45: cluster of differentiation antigen 45; CD8: cluster of differentiation antigen 8; CD4: cluster of differentiation antigen 4; CD3ε: cluster of differentiation antigen 3 epsilon chain; CD3γ: cluster of differentiation antigen 3 gamma chain; CD3δ: cluster of differentiation antigen 3 delta chain; Cd3ζ: cluster of differentiation antigen 3 zeta chain; TCRα: T cell receptor alpha chain; TCRβ: T cell receptor beta chain; CD40L: cluster of differentiation antigen 40 ligand; ICOS: inducible T-cell co-stimulator; CD28: cluster of differentiation antigen 28; SHP1: protein-tyrosine phosphatase-1; LCK: lymphocyte-specific protein tyrosine kinase; FYN: proto-oncogene tyrosine-protein kinase Fyn; LAT: linker for activation of T cells; GRB2: growth factor receptor-binding protein 2; SOS: son of sevenless; PI3K: phosphatidylinositol-4,5-bisphosphate 3-kinase; CBL: Casitas B-lineage lymphoma; ZAP70: zeta-chain (TCR) associated protein kinase; GADS: GRB2-related adaptor protein 2; PCL-γ1: phospholipase C gamma 1; PKCθ: protein kinase C theta; PDK1: pyruvate dehydrogenase kinase isoform 1; AKT: Rac serine/threonine-protein kinase; SLP-76: SH2 domain-containing leukocyte protein of 76 kDa; IKT: IL2-inducible T-cell kinase; RasGRP1: Ras guanyl-releasing protein 1; TAK1: TGF-beta activated kinase 1; CARMA1: caspase recruitment domain family, member 11; BCL-10: B cell lymphoma 10; MALT1: mucosa associated lymphoid tissue lymphoma translocation gene; COT: cancer Osaka thyroid oncogene; GSK-3β: glycogen synthase kinase 3 beta; NCK: NCK adaptor protein 1; VAV: vav guanine nucleotide exchange factor; Ras: GTPase HRas; MKK7: mitogen-activated protein kinase kinase 7; Dlgh1: disks large protein 1; NIK: NF-kappa-beta-inducing kinase; P38: p38 MAP kinase; PAK: p21-activated kinase 1; Rho/Cdc42: cell division control protein 42; Raf: Raf proto-oncogene serine/threonine-protein kinase; JNK2: c-Jun N-terminal kinase 2; IKKα: inhibitor of nuclear factor kappa-B kinase subunit alpha; IKKβ: inhibitor of nuclear factor kappa-B kinase subunit beta; IKKγ: inhibitor of nuclear factor kappa-B kinase subunit gamma; MEK2: mitogen-activated protein kinase kinase 2; IκB: NF-kappa-B inhibitor; NF-κB: nuclear factor NF-kappa-B; ERK: extracellular signal-regulated kinase; NFAT: nuclear factor of activated T-cells; AP1: activator protein-1.

During their development, T-cells differentiate into either CD4^+^ helper or CD8^+^ cytolytic T cells [60]. Antigen-presenting cells expose antigenic peptides to T lymphocytes through the major histocompatibility complex (MHC) molecules [61]. CD4^+^ lymphocytes have the function of collaborating on the activation of other immune system cells, as well as recruiting them to sites of infection [62]. CD8^+^ T lymphocytes present cytotoxic capability, eliminating cells infected with viruses, intracellular bacteria and parasites as well as tumor cells [63]. The present turbot pyrosequencing effort provided several contigs similar to proteins intervening in the cascade of T lymphocyte activation (Figure 10). As occurred with the B cell receptor signaling pathway, the previous information for this activation cascade was very scarce in *S. maximus* and was limited to few proteins. An additional table containing information about contigs and singletons showing homology to molecules involved in the T cell receptor signaling pathway is provided (Table S6).

#### Apoptosis or programmed cell death

**Figure 11:**
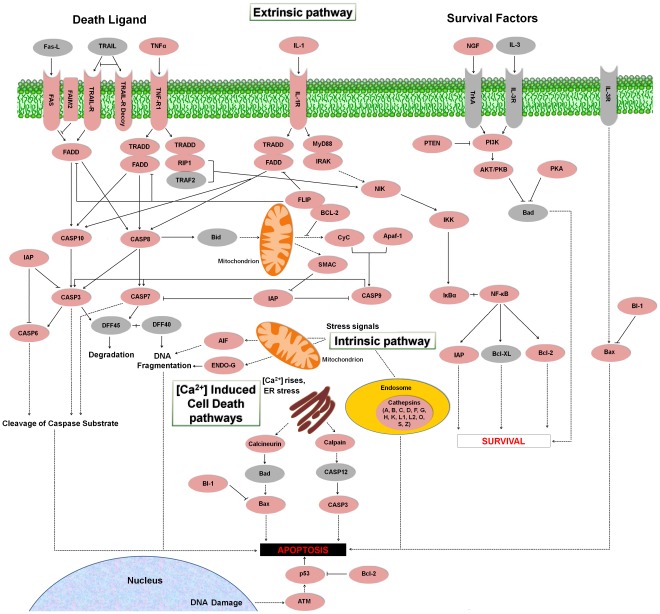
Apoptosis cascade representing the present and absent proteins in the pyrosequencing results. Proteins appearing in the turbot transcriptome are represented in reddish color and absent proteins in grey color. Fas-L: Fas ligand; TRAIL: TNF-related apoptosis-inducing ligand; TNFα: Tumor necrosis factor alpha; IL-1: interleukin 1; NGF: nerve growth factor; IL-3: interleukin 3; Fas: tumor necrosis factor receptor superfamily member 6; FAIM2: Fas apoptotic inhibitory molecule 2; TRAIL-R: tumor necrosis factor receptor superfamily member 10; TRAIL-R Decoy: TRAIL receptor with a truncated death domain; TNF-R1: tumor necrosis factor receptor superfamily member 1A; IL-1R: interleukin 1 receptor; TrkA: neurotrophic tyrosine kinase receptor type 1; IL-3R: interleukin 3 receptor; FADD: FAS-associated via death domain; TRADD: TNF receptor superfamily 1alpha-associated via death domain; MyD88: myeloid differentiation primary response protein MyD88; PTEN: Phosphatase and tensin homolog; PI3K: phosphatidylinositol-4,5-bisphosphate 3-kinase; RIP1: receptor-interacting serine/threonine-protein kinase 1; IRAK: interleukin-1 receptor-associated kinase; TRAF2: TNF receptor-associated factor 2; AKT: TNF receptor-associated factor; PKB: protein kinase B; PKA: protein kinase A; NIK: NF-kappa-beta-inducing kinase; FLIP: FADD-like apoptosis regulator; BCL-2: B cell lymphoma 2; IKK: inhibitor of nuclear factor kappa-B kinase; Bad: BCL2-antagonist of cell death; CASP3: caspase 3; CASP6: caspase 6; CASP7: caspase 7; CASP8: caspase 8; CASP9: caspase 9; CASP10: caspase 10; CASP12: caspase 12; Bid: BH3 interacting domain death agonist; CyC: cytochrome c; Apaf-1: apoptotic protease-activating factor; IAP: inhibitor of apoptosis protein; SMAC: second mitochondria-derived activator of caspases; IκBα: NF-kappa-B inhibitor alpha; NF-κB: nuclear factor NF-kappa-B; DFF45: DNA fragmentation factor of 45 kD; DFF40: DNA fragmentation factor of 40 kD; BI-1: Bax inhibitor-1; AIF: apoptosis-inducing factor; ENDO-G: endonuclease G; Bcl-XL: B-cell lymphoma-extra large; Bax: Bcl-2-associated X protein; p53: tumor protein p53; ATM: ataxia telangectasia mutated family protein.

Apoptosis is an essential mechanism for the development and survival of organisms. Unwanted, damaged or infected cells are removed involving numerous molecules [64]. Caspases are proteases with a pivotal role in apoptosis among other functions [65]. Our transcriptome analysis showed for the first time that the vast majority of central molecules that control programmed cell death in higher vertebrates, presented homologues in the flatfish *S. maximus*, suggesting the existence of a phylogenetically conserved apoptotic machinery (Figure 11). An additional table containing information about contigs and singletons showing homology to molecules implicated in the apoptosis is also provided (Table S7).

#### Cytokines

Cytokines are cell-signalling proteins involved in haematopoiesis, inflammation and defense against pathogens [66]. Major cytokines include interleukins (ILs), chemokines, interferons (IFNs), tumor necrosis factors (TNFs), colony stimulating factors (CSFs) and growth factors (GFs) [67]. Among the interleukins, our results showed the presence of putative contigs for IL-1β, IL-8, IL-11, IL-12β, IL-15, IL-16, IL-18 and IL-25. Chemokines are characterized by cysteine residues and separated into two groups depending on the presence (C-X-C family) or absence (CC family) of an intervening amino acid between cysteine residues [68]. Several contigs and singletons were identified as CC and C-X-C motif chemokines. For the IFNs group, there was a unique contig presenting homology to *D. rerio* IFN phi 2 (IFNΦ2) and a singleton annotated as IFN interferon alpha 2 precursor [Salmo salar]. TNFα, a pleiotropic pro-inflammatory cytokine produced by numerous immune cells during inflammation [69], appeared also in our collection, as well as TNFβ. Among the CSFs, which regulate the production and functional activities of hematopoietic cells [70], we have found four types: macrophage colony-stimulating factor 1 (MCSF-1) and 2 (MCSF-2), granulocyte colony-stimulating factor 1 (GCSF-1) and the megakaryocyte colony-stimulating factor thrombopoietin. Some growth factors appearing in the turbot transcriptome were fibroblast growth factor (FGF), transforming growth factor (TGF), bone morphogenetic protein (BMP), epidermal growth factor (EGF), platelet-derived growth factor (PDGF), vascular endothelial growth factor (VEGF), nerve growth factor (NGF) and hepatocyte growth factor or hematopoietin (HGF). Moreover, we found several contigs presenting homology to cytokine receptors and cytokine-related proteins.

#### Other immune molecules

Concerning other pattern recognition receptors (PRRs) different to TLRs, lectins are a family of carbohydrate-recognition proteins and they have been implicated in the direct first-line defense against pathogens and immune regulation [23]. The turbot transcriptome exhibited abundant lectins, especially C-type lectins. However, other lectins were also detected, such as S-type lectins (Galectins 1, 2, 4, 8 and 9) and L-rhamnose binding lectins. With regards to β-glucan recognition proteins, only one contig was found with homology to β-1,3-glucan-binding protein 1 (BGBP-1). Moreover, peptidoglycan recognition proteins (PGRPs) and only one contig for lipopolysaccharide-binding protein (LBP) were detected.

Among other immune-related proteins, it is worth pointing out the identification of several protease inhibitors, antimicrobial peptides and other proteins with cytolytic activity (hepcidin, defensin, antimicrobial peptide precursor, Nk-lysin, lysozyme G and C, perforin-1, granzyme and other serine proteases) and also several heat shock proteins (Hsp10, Hsp16, Hsp20, Hsp27, Hsp40, Hsp60, Hsp68, Hsp70, Hsp71, Hsp75, Hsp83 and Hsp90).

Furthermore, there was a large representation of iron-binding proteins. Hemoglobin, ferritin, transferrin, serotransferrin, haptoglobin and hemopexin were the most represented. As was mentioned above, viruses need an iron-replete host for an efficient replication and they can alter the levels of proteins involved in iron homeostasis. Moreover, hemorrhages generated during infections, especially produced by VHSV, could influence the expression of these molecules.

### Conclusions

Comparison of our results with previous transcriptomic studies from *S. maximus* using conventional Sanger sequencing confirms the efficacy of 454-pyrosequencing to improve the genomic knowledge on this flatfish, revealing a large number of contigs. We have obtained 55,404 contigs containing at least 2 reads and 181,845 singletons. Approximately 45% of the contigs were successfully annotated and 21.21% of the singletons submitted to Blast2GO presented homology to proteins deposited in the databases. The wealth of information generated by pyrosequencing in *S. maximus* provides a new resource for future investigations in this economically important species, including improvement of microarray tools and identification of genetic markers. Moreover, we have identified several thousand contigs and singletons with a potential immune function and observed that the majority of proteins involved in the main immune-pathways in humans have also its putative homologues in turbot.

## Materials and Methods

### Turbot Sampling

Fifty-two juvenile turbots (average weight 36 g) were obtained from a commercial fish farm in Galicia (NW Spain). Animals were acclimated to laboratory conditions for 2 weeks, maintained at 18°C and fed daily with a commercial diet. The fish were divided into one batch of 4 individuals and four batches of 12 individuals each one. Turbots from the batch with 4 individuals were challenged by intramuscular injection with 100 µl of a Nodavirus (10^7^ TCID_50_/fish) suspension (strain AH95-NorA) [71]. The first batch of 12 individuals was challenged by intraperitoneal injection with 100 µl of VHSV (4 x 10^9^ TCID_50_/fish) suspension (strain UK-860/94) [72]. The second batch was intramusculary injected with pMCV 1.4 (Ready-Vector, Madrid, Spain) (2 µg/fish). pMCV 1.4 is an expression vector typically used for the construction of DNA vaccines and empty pMCV 1.4 served as a double-stranded DNA viral stimulus. The third batch was challenged with pMCV 1.4-G_860_ (2 µg/fish), which codes the G glycoprotein from VHSV UK-860/94 strain (GenBank accession AY546628) and was used as a DNA vaccine. The fourth batch was intraperitoneally stimulated with poly I:C (0.1 mg/fish) (SIGMA) mimicking a viral infection. Viral suspensions were prepared using minimal essential medium (MEM, GIBCO) supplemented with 2% fetal bovine serum (FBS), penicillin (100 IU/ml) (Invitrogen) and streptomycin (100 µg/ml) (Invitrogen). Poly I:C and plasmid constructs were inoculated in 1x Phosphate Buffered Saline (PBS 1x).

In order to obtain mRNA representative of both innate and adaptive immune systems comprising the full spectrum of the immune response, gill, liver, spleen and head kidney tissues were removed from 2 individuals for each sampling point (1 h, 6 h, 24 h, 3 days, 7 days and 14 days post-inoculation) for the batches with 12 individuals. For the batch of fish infected with Nodavirus, the brain, as target organ, was removed from 2 individuals at 24 h and 3 days after injection. We have used only 2 time points (1 and 3 days) for Nodavirus infection since previous works in other fish species [73], [74] showed that the up-regulation of several immune-relevant genes begins at 24 hours post-infection and reached their highest levels at 3 days after intramuscular infection. This could suggest that immune responses are activated when the viruses reach their target organ (brain) and start their replication [73]. For each sampling time and treatment, equal amounts of the same tissue from the two fish were pooled.

Healthy turbot larvae at early developmental stages (until 1 month) were also taken in order to detect immune-related genes highly expressed during the ontogenic development. Turbot larvae were pooled in one sample. All samples were stored at −80 °C in 500 µl of *TRIzol®* (Invitrogen) until RNA isolation.

### RNA Isolation, cDNA Production and Pyrosequencing

Total RNA was extracted from pooled tissues using *TRIzol®* (Invitrogen) in accordance with instructions provided by the manufacturer in combination with the RNeasy mini kit (Qiagen) for RNA purification after DNase treatment (RNase-free DNase set, Qiagen). Quantity of total RNA purified from each pool was determined using the spectrophotometer Nanodrop ND-100. RNA from each organ and sampling point was pooled by mixing 1 µg of RNA from each type of stimuli. The final concentration was adjusted to 1 µg/µl. RNA quality was assessed by electrophoresis on a denaturing agarose gel (1X) in MOPS buffer in order to visualize the 18S and 28S ribosomal RNA bands. RNA integrity was furthermore investigated using the Bioanalyzer 2100 (Agilent Technologies).

RNA extracted from stimulated/infected *S. maximus* was used as the source of starting material for cDNA synthesis Full-length-enriched double stranded cDNA was synthesized from 1,5 µg of pooled total RNA using MINT cDNA synthesis kit (Evrogen, Moscow, Russia) according to manufacturer’s protocol, and was subsequently purified using the QIAquick PCR Purification Kit (Qiagen USA, Valencia, CA). The amplified cDNA was normalized using Trimmer kit (Evrogen, Moscow, Russia) to minimize differences in representation of transcripts. The method involves denaturation-reassociation of cDNA, followed by a digestion with a Duplex-Specific Nuclease (DSN) enzyme [75], [76]. The enzymatic degradation occurs primarily on the highly abundant cDNA fraction. The single-stranded cDNA fraction was then amplified twice by sequential PCR reactions according to the manufacturer’s protocol. Normalized cDNA was purified using the QIAquick PCR Purification Kit (Qiagen USA, Valencia, CA).

500 ng of normalized cDNA were used to generate a 454 library. cDNA was fractionated into small, 300- to 800-basepair fragments and the specific A and B adaptors were ligated to both the 3' and 5' ends of the fragments. The A and B adaptors were used for purification, amplification, and sequencing steps. One sequencing run was performed on the GS-FLX using Titanium chemistry. 454 Sequencing is based on sequencing-by-synthesis, addition of one (or more) nucleotide(s) complementary to the template strand results in a chemiluminescent signal recorded by the CCD camera within the instrument. The signal strength is proportional to the number of nucleotides incorporated in a single nucleotide flow. All reagents and protocols used were from Roche 454 Life Sciences, USA. RNA was normalized, processed and sequenced by the Unitat de Genòmica (CCiT-UB, Barcelona, Spain).

**Figure 12:**
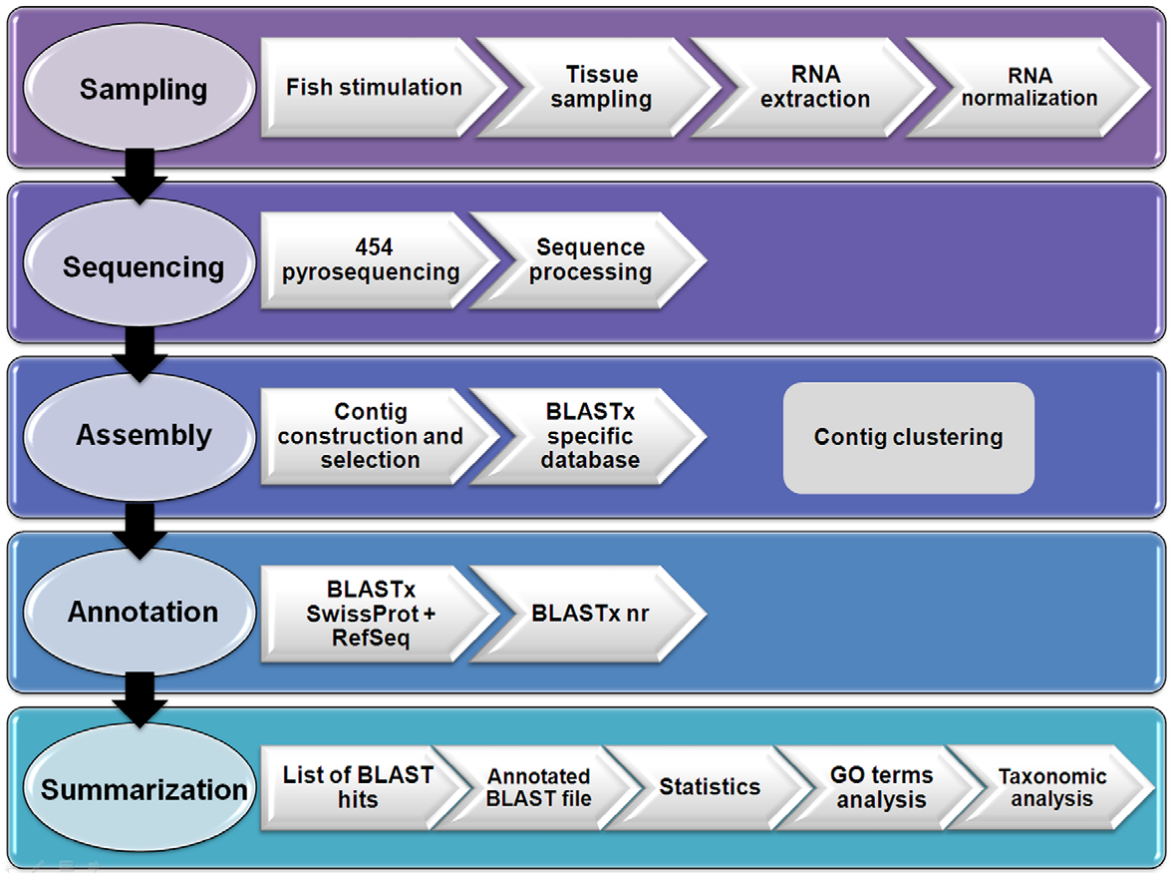
Flowchart representing the data processing pipeline for *de novo* transcriptome assembly and annotation of *Scophthalmus maximus*.

### Assembly, Functional Annotation and Comparative Analysis

Pyrosequencing raw data has been processed with the Roche quality control pipeline using default settings. Seqclean software (http://compbio.dfci.harvard.edu/tgi/software/) was used in order to process all sequences prior to assembly. Sequences shorter than 50 bp were discarded and reads with at least 18 bp and 95% identity with a contaminant vector were considered invalid. Then A homopolymers and adapters from cDNA library construction were trimmed. Sequences were submitted to MIRA V3.2.0 [77] for assembling the transcriptome ensuring a minimum left clip on each read for the first 4 nucleotides in order to eliminate possible biases introduced by RNA normalization that could not be fully removed by the cleaning process and only those clean reads presenting a minimum length of 40 bp were assembled. *Blastclust* program [78], one part of BLAST package from NCBI, was used on the full fasta contig file to group similar contigs into clusters (groups of transcripts from the same gene) if at least 60% of the positions is equal or over 95% identity. Singletons shorter than 100 bp were removed and the remaining sequences (about 100,000) were submitted together with the contigs to *Blastclust* for identifying the singletons which do not cluster with the contigs. A total of 64,704 singletons were selected for the annotation step.

The annotation of *S. maximus* consensus contigs and singletons not clustering with contigs was carried out by *Blastx* search [78] against the Swissprot and Metazoan RefSeqs databases. The non-annotated sequences using these databases were submitted to GenBank non-redundant (nr) database. In both cases the E-value cut-off was 1*e^−^* ^03^. The largest contig belonging to each cluster was submitted to *Blast2GO* software [49], [50] in order to predict the functions of the sequences and assign Gene Ontology terms. The same sequences of turbot were compared to the ESTs and nucleotide sequences of zebrafish (*D. rerio*) and Japanese flounder (*P. olivaceus*) deposited in the NCBI databases using BLASTn algorithm. For *D. rerio* there were 1,488,275 ESTs and 72,554 unique nucleotide sequences (based on GeneInfo identifiers) and for *P. olivaceus* there were 15,234 and 1,936, respectively. The E-value cut-off was 1*e^−^* ^05^. With the aim of illustrating the outline flow for the transcriptome annotation procedure a diagram is shown in Figure 12.

### Identification of Immune-related Proteins

GO terms at level 2, 3 and 4 having a direct relationship with immunity were used for selecting putative immune-related proteins together with a thorough analysis conducted in our laboratory based on an extensive list of immune terms and a comprehensive literature review.

In order to establish the presence and absence of proteins belonging to the more relevant immune-pathways in our pyrosequencing results, we have used the KEGG reference pathways [79] as a template for constructing the following immune-cascades by hand: Complement pathway, Toll-like receptor signaling pathway, B cell receptor signaling pathway, T cell receptor signaling pathway and Apoptosis cascade. Additional molecules were included in some cases after bibliographic review.

## Supporting Information

Table S1 **Sequence of the singletons submitted to Blast2GO for annotation.**
(XLSX)

Table S2 **Molecular function assignment (3^rd^ level GO terms) for the transcriptomic sequences (contigs) of** ***Scophthalmus maximus.***
(DOCX)

Table S3 **Information of the contigs and singletons identified as homologous to molecules involved in the Complement pathway.**
(XLSX)

Table S4 **Information of the contigs and singletons identified as homologous to molecules involved in the Toll-like receptor signaling pathway.**
(XLSX)

Table S5 **Information of the contigs and singletons identified as homologous to molecules involved in the B cell receptor signaling pathway.**
(XLSX)

Table S6 **Information of the contigs and singletons identified as homologous to molecules involved in the T cell receptor signaling pathway.**
(XLSX)

Table S7 **Information of the contigs and singletons identified as homologous to molecules involved in the Apoptosis or programmed cell death.**
(XLSX)
