# Supplementary material for: High-Throughput Sequence Analysis of Turbot (Scophthalmus maximus) Transcriptome Using 454-Pyrosequencing for the Discovery of Antiviral Immune Genes
Source: PLoS One. 2012 May 18;7(5):e35369. doi: 10.1371/journal.pone.0035369 (PMC3356354; doi:10.1371/journal.pone.0035369)
Supplement: Table S2 — Molecular function assignment (3rd level GO terms) for the transcriptomic sequences (contigs) of Scophthalmus maximus. (DOCX) [file pone.0035369.s002.docx]

**Table S2. Molecular function assignment (3^rd^ level GO terms) for the transcriptomic sequences (contigs) of *Scophthalmus maximus***

| **Molecular Function (3^rd^ level)** | **Number of contigs** | | **Percentage (%)** |
| --- | --- | --- | --- |
| Protein binding | | 6813 | 19.62 |
| Iron binding | | 3680 | 10.598 |
| Hydrolase activity | | 3587 | 10.33 |
| Nucleic acid binding | | 2432 | 7.004 |
| Nucleotide binding | | 2241 | 6.454 |
| Transferase activity | | 2005 | 5.774 |
| Nucleoside binding | | 1506 | 4.337 |
| Carbohydrate binding | | 1502 | 4.326 |
| Substrate-specific transporter activity | | 1400 | 4.032 |
| Oxidoreductase activity | | 1252 | 3.606 |
| Signal transducer activity | | 1109 | 3.194 |
| Enzyme inhibitor activity | | 1027 | 2.958 |
| Lipid binding | | 872 | 2.511 |
| Transmembrane transporter activity | | 852 | 2.454 |
| Pattern binding | | 488 | 1.405 |
| Ligase activity | | 423 | 1.218 |
| Transcription cofactor activity | | 420 | 1.210 |
| Enzyme activator activity | | 314 | 0.904 |
| Cell surface binding | | 301 | 0.867 |
| Lyase activity | | 266 | 0.766 |
| Nucleoside-triphosphatase regulator activity | | 236 | 0.680 |
| Isomerase activity | | 217 | 0.625 |
| Cofactor binding | | 186 | 0.536 |
| Carboxylic acid binding | | 149 | 0.429 |
| Peptide binding | | 121 | 0.348 |
| Kinase regulator activity | | 117 | 0.337 |
| Ribonucleoprotein binding | | 110 | 0.317 |
| RNA polymerase II transcription factor activity | | 105 | 0.302 |
| Transcription repressor activity | | 103 | 0.297 |
| Peroxidase activity | | 102 | 0.294 |
| Metal cluster binding | | 84 | 0.242 |
| Amine binding | | 68 | 0.196 |
| Drug binding | | 64 | 0.184 |
| Calcium channel regulator activity | | 55 | 0.158 |
| Phosphatase regulator activity | | 50 | 0.144 |
| Vitamin binding | | 48 | 0.138 |
| Transcription activator activity | | 45 | 0.13 |
| Translation regulator activity. nucleic acid binding | | 43 | 0.124 |
| Antigen binding | | 37 | 0.107 |
| Nucleobase binding | | 30 | 0.086 |
| Channel inhibitor activity | | 24 | 0.069 |
| Small protein activating enzyme activity | | 23 | 0.066 |
| Extracellular matrix binding | | 22 | 0.063 |
| Cyclase activity | | 19 | 0.055 |
| Transcription elongation regulator activity | | 19 | 0.055 |
| Chromatin binding | | 19 | 0.055 |
| Alcohol binding | | 19 | 0.055 |
| Cofactor transporter activity | | 18 | 0.052 |
| Demethylase activity | | 18 | 0.052 |
| Neurotransmitter binding | | 16 | 0.046 |
| Hormone binding | | 16 | 0.046 |
| Translation repressor activity | | 10 | 0.029 |
| Protein complex scaffold | | 9 | 0.026 |
| Lipopolysaccharide binding | | 7 | 0.020 |
| Structural constituent of cuticle | | 7 | 0.020 |
| Cyclase regulator activity | | 5 | 0.014 |
| Extracellular matrix structural constituent | | 5 | 0.014 |
| Transcription terminator factor activity | | 3 | 0.009 |
| Transcription factor activity | | 2 | 0.006 |
| Molecular adaptor activity | | 2 | 0.006 |
| Oxygen binding | | 1 | 0.003 |
